# Supplementary material for: Light modulates task-dependent thalamo-cortical connectivity during an auditory attentional task
Source: Commun Biol. 2023 Sep 16;6:945. doi: 10.1038/s42003-023-05337-5 (PMC10504287; doi:10.1038/s42003-023-05337-5)
Supplement: Supplementary file 3 — Reporting Summary [file 42003_2023_5337_MOESM3_ESM.pdf]

nature portfolio

Corresponding author(s): Gilles Vandewalle

Last updated by author(s): 31/08/2023

Reporting Summary

Nature Portfolio wishes to improve the reproducibility of the work that we publish. This form provides structure for consistency and transparency in reporting. For further information on Nature Portfolio policies, see our Editorial Policies and the Editorial Policy Checklist.

Statistics

For all statistical analyses, confirm that the following items are present in the figure legend, table legend, main text, or Methods section.

n/a	Confirmed	
☐	☒	The exact sample size (*n*) for each experimental group/condition, given as a discrete number and unit of measurement
☐	☒	A statement on whether measurements were taken from distinct samples or whether the same sample was measured repeatedly
☐	☒	The statistical test(s) used AND whether they are one- or two-sided<br>*Only common tests should be described solely by name; describe more complex techniques in the Methods section.*
☒	☐	A description of all covariates tested
☒	☐	A description of any assumptions or corrections, such as tests of normality and adjustment for multiple comparisons
☐	☒	A full description of the statistical parameters including central tendency (e.g. means) or other basic estimates (e.g. regression coefficient) AND variation (e.g. standard deviation) or associated estimates of uncertainty (e.g. confidence intervals)
☒	☐	For null hypothesis testing, the test statistic (e.g. *F*, *t*, *r*) with confidence intervals, effect sizes, degrees of freedom and *P* value noted<br>*Give P values as exact values whenever suitable.*
☒	☐	For Bayesian analysis, information on the choice of priors and Markov chain Monte Carlo settings
☐	☒	For hierarchical and complex designs, identification of the appropriate level for tests and full reporting of outcomes
☐	☒	Estimates of effect sizes (e.g. Cohen's *d*, Pearson's *r*), indicating how they were calculated

Our web collection on statistics for biologists contains articles on many of the points above.

Software and code

Policy information about availability of computer code

Data collection	OpenSesame<br>MR-compatible headphones (Sensimetrics, Malden, MA)<br>response box (Current Designs, Philadelphia, PA)<br>LED light source (SugarCUBE, Cypress, California)<br>optic fiber (Setra, Boxborough, Massachusetts)<br>filter wheel (Spectral Products, AB300)<br>eye tracking system (EyeLink 1000Plus, SR-Research, Ottawa Canada)<br>MAGNETOM Terra 7T MRI system (Siemens Healthineers, Erlangen, Germany)
Data analysis	Statistical Parametric Mapping 12 (SPM12; https://www.fil.ion.ucl.ac.uk/spm/software/spm12/)<br>Matlab R2019 (MathWorks, Natick, Massachusetts)<br>Advanced Normalization Tools (ANTs, Penn Image Computing and Science Laboratory, UPenn, USA, http://stnava.github.io/ANTs)<br>RObust Brain EXtraction (ROBEX, https://www.nitrc.org/projects/robex)<br>FMRIB Software Library (FSL, Analysis Group, Oxford University, UK, https://fsl.fmrib.ox.ac.uk/fsl/fslwiki)<br>PhysIO Toolbox (Translational Neuromodeling Unit, ETH Zurich, Switzerland)<br>Parametrical Empirical Bayes (PEB) approach as implemented in Dynamic Causal Modelling (DCM) in SPM12

For manuscripts utilizing custom algorithms or software that are central to the research but not yet described in published literature, software must be made available to editors and reviewers. We strongly encourage code deposition in a community repository (e.g. GitHub). See the Nature Portfolio guidelines for submitting code & software for further information.

Data

Policy information about availability of data

All manuscripts must include a data availability statement. This statement should provide the following information, where applicable:

- Accession codes, unique identifiers, or web links for publicly available datasets
- A description of any restrictions on data availability
- For clinical datasets or third party data, please ensure that the statement adheres to our policy

Statistical Parametric Mapping 12 (SPM12; https://www.fil.ion.ucl.ac.uk/spm/software/spm12/)
Matlab R2019 (MathWorks, Natick, Massachusetts)
Advanced Normalization Tools (ANTs, Penn Image Computing and Science Laboratory, UPenn, USA, http://stnava.github.io/ANTs)
RObust Brain EXtraction (ROBEX, https://www.nitrc.org/projects/robex)
FMRIB Software Library (FSL, Analysis Group, Oxford University, UK, https://fsl.fmrib.ox.ac.uk/fsl/fslwiki)
PhysIO Toolbox (Translational Neuromodeling Unit, ETH Zurich, Switzerland)
Parametrical Empirical Bayes (PEB) approach as implemented in Dynamic Causal Modelling (DCM) in SPM12

Research involving human participants, their data, or biological material

Policy information about studies with human participants or human data. See also policy information about sex, gender (identity/presentation), and sexual orientation and race, ethnicity and racism.

Reporting on sex and gender	Twenty subjects were recruited for the study. Nineteen participants were considered for further analysis (24.05, SD 2.63; 12 women) after excluding a participant for poor performance.
Reporting on race, ethnicity, or other socially relevant groupings	NA
Population characteristics	All participants provided informed consent to participate in the study and none reported a history of ophthalmic disorders. A semi-structured interview and several questionnaires assessed exclusion criteria, which were as follows: body mass index > 25, clinical level of depression and anxiety, addiction or diagnosed psychiatric disorders; having worked night shift during the last year or having travelled through more than one time zone during the last two months, smoking, use of psychoactive drugs, excessive caffeine and alcohol consumption (i.e., >4 caffeine units/day; >14 alcohol units/week), being pregnant or at risk of pregnancy. The 21 Item Beck Anxiety and Depression Inventory II, the Pittsburgh Sleep Quality Index, the Epworth Sleepiness Scale, the Horne-Östberg Munich chronotype questionnaire, and the Seasonal Pattern Assessment Questionnaire were used to assess mood, sleep quality, daytime sleepiness, chronotype and changes in mood behavior respectively.
Recruitment	Participants were recruited through advertisements on local journals and the University of Liège website and via emails to students or member of the staff at the University.
Ethics oversight	The study was approved by the Ethical Committee of the University of Liège and participants received a financial compensation.

Note that full information on the approval of the study protocol must also be provided in the manuscript.

Field-specific reporting

Please select the one below that is the best fit for your research. If you are not sure, read the appropriate sections before making your selection.

☒ Life sciences ☐ Behavioural & social sciences ☐ Ecological, evolutionary & environmental sciences

For a reference copy of the document with all sections, see nature.com/documents/nr-reporting-summary-flat.pdf

Life sciences study design

All studies must disclose on these points even when the disclosure is negative.

Sample size	Twenty healthy young adults were recruited. Optimal sensitivity and power analyses in MRI/DCM/PEB remains under investigation [e.g. REF1]. We nevertheless computed a prior sensitivity analysis to get an indication of the minimum detectable effect size in our main analyses given our sample size. According to G*Power 3 (version 3.1.9.4) REF2 taking into account a power of .8, an error rate α of .025 (corrected for 2 tests), a sample size of 20 allowed us to detect large effect sizes r > .5 (lower limit of large effect size; 1-sided; absolute values; confidence interval: 0.07, 0.77; R^2 > .25, R^2 confidence interval: .005 – .59) within a linear multiple regression framework including 1 predictor. Based on this and on prior literature (REF3) we deemed the sensitivity reasonable.<br><br>REF1: Lombardo, M. V., et al. Improving effect size estimation and statistical power with multi-echo fMRI and its impact on understanding the neural systems supporting mentalizing. Neuroimage, 142, 55-66 (2016).<br>REF2: Faul, F., et al. Statistical power analyses using G*Power 3.1: Tests for correlation and regression analyses. Behav. Res. Methods 41, 1149–1160 (2009).

REF3 : Vandewalle, G., et al. Daytime light exposure dynamically enhances brain responses. Current Biology, 16(16), 1616-1621 (2006).

Data exclusions

One participant did not correctly follow the task assignment (accuracy < 3SD from the group mean). His data were therefore excluded, and nineteen participants were considered for further analysis.

Replication

Our effective connectivity findings are replicated on both hemispheres (tested separately).

Randomization

NA

Blinding

NA

Reporting for specific materials, systems and methods

We require information from authors about some types of materials, experimental systems and methods used in many studies. Here, indicate whether each material, system or method listed is relevant to your study. If you are not sure if a list item applies to your research, read the appropriate section before selecting a response.

Materials & experimental systems

n/a	Involved in the study	
☒	☐	Antibodies
☒	☐	Eukaryotic cell lines
☒	☐	Palaeontology and archaeology
☒	☐	Animals and other organisms
☒	☐	Clinical data
☒	☐	Dual use research of concern
☒	☐	Plants

Methods

n/a	Involved in the study	
☒	☐	ChIP-seq
☒	☐	Flow cytometry
☐	☒	MRI-based neuroimaging

Magnetic resonance imaging

Experimental design

Design type

Task functional MRI

Design specifications

Participants had to detect rare (20%) deviant tones (100Hz; 500ms) presented pseudo-randomly within a stream of more frequent (80%) standard (500Hz; 500ms) tones (interstimulus interval: 2s). A short procedure preceding the task ensured optimal auditory perception of both stimuli. While performing the task, participants were exposed to 30s-blocks of active, blue-enriched cold polychromatic light (6500K; 92 melanopic EDI lux) meant to recruit ipRGC photoreception, or control orange monochromatic light (5.28x1012photons/cm2/s; 590nm, 10nm at full width half maximum; 0.16 melanopic EDI lux) meant to trigger a visual response while recruiting much less ipRGCs. Light periods were separated by ~15s darkness periods (<0.01 lux). Seven blocks of each light were administered and a total of 250 standard and 63 deviant tones were delivered. The deviant tones were equally distributed across the three light blocks/ conditions. The oddball task lasted for around 12 minutes.

Behavioral performance measures

Behavioral performance was not the primary outcome of the study. Nevertheless, we computed participants' overall performance, which was high (mean 0.96 ± 0.005), and their reaction times (RTs) to the deviant tones, which were not influenced by the light condition (t(19)= 2.10, p= 0.47, Cohen's d= 0.05).

Acquisition

Imaging type(s)

Functional

Field strength

7T

Sequence & imaging parameters

Structural and functional MRI data were acquired using a MAGNETOM Terra 7T MRI system (Siemens Healthineers, Erlangen, Germany) with a 32-channel receiver and 1 channel transmit head coil (Nova Medical, Wilmington, MA, USA). To improve uniformity of the B1 radio frequency excitations, dielectric pads were placed between the head of the subjects and the receiver coil (Multiwave Imaging, Marseille, France). Multislice T2*-weighted fMRI images were obtained with a multi-band Gradient-Recalled Echo - Echo-Planar Imaging (GRE-EPI) sequence using axial slice orientation (TR = 2340 ms, TE = 24 ms, FA = 90°, no interslice gap, in-plane FoV = 224 mm × 224 mm, matrix size = 160 × 160 × 86, voxel size = 1.4 × 1.4 × 1.4 mm3). The three initial scans were discarded to avoid saturation effects. For anatomical imaging, a high-resolution T1-weighted image was acquired using a Magnetization-Prepared with 2 RApid Gradient Echoes (MP2RAGE) sequence: TR = 4300 ms, TE = 1.98 ms, FA = 5°/6°, TI = 940ms/2830 ms, bandwidth = 240 Hz, matrix size = 256x256, 224 axial slices, acceleration factor = 3, voxel size = (0.75x0.75x0.75) mm3. Participants' pulse and respiration were also recoded to subsequently correct for physiological noise in the fMRI data.

Area of acquisition

Whole brain

Diffusion MRI ☐ Used ☒ Not used

Preprocessing

Preprocessing software	Statistical Parametric Mapping 12 (SPM12; https://www.fil.ion.ucl.ac.uk/spm/software/spm12/) under Matlab R2019 (MathWorks, Natick, Massachusetts)<br>Advanced Normalization Tools (ANTs, Penn Image Computing and Science Laboratory, UPenn, USA, http://stnava.github.io/ANTs) or RObust Brain EXtraction (ROBEX, https://www.nitrc.org/projects/robex)<br>FMRIB Software Library (FSL, Analysis Group, Oxford University, UK, https://fsl.fmrib.ox.ac.uk/fsl/fslwiki)
Normalization	Data were normalized prior to second level analysis
Normalization template	Brain extracted T1-images were used to create a T1-weighted group template using ANTs. For each subject, first level analysis was performed in their native space (non-registered EPIs were used for statistical test) to prevent any possible error that may be caused by coregistration. Prior to second level analysis, all statistical maps obtained from the first level analysis, were first transferred to the group template space and then the MNI space (1x1x1mm3). All the registrations were performed with ANTs.
Noise and artifact removal	SPM12 was used to remove high-intensity background noise, automatically reorient, and correct structural images for intensity bias. Brains were extracted to ensure optimal coregistration. For functional volumes, voxel-displacement maps were computed using the phase and magnitude images. "Realign & Unwarp" was then applied to the EPI images to correct for head motion and for static and dynamic susceptibility induced variance. Realigned and distortion corrected EPI images underwent brain extraction and the final images were smoothed with a Gaussian kernel characterized by a full width at half maximum of 3 mm.
Volume censoring	Movement parameters, as well as cardiac and respiratory parameters computed with the PhysIO Toolbox (Translational Neuromodeling Unit, ETH Zurich, Switzerland), were included as regressors of no interest. Low-frequency drifts were removed by using high-pass filtering with a cut-off period of 128 s.

Statistical modeling & inference

Model type and settings	Univariate analysis: The contrast of interest of the univariate analyses consisted in the main effect of deviant tones. Summary statistic images resulting from linear contrasts (in MNI space) were entered in a second-level analysis accounting for intersubject variance and corresponding to a one-sample t test for brain responses to deviant sounds. Results were corrected for multiple comparisons at the voxel level ($p < 0.05$) through a false discovery rate procedure.<br><br>Effective connectivity: we used the Dynamic Causal Modelling (DCM) framework as implemented in SPM12. Three inputs were specified in a design matrix then imported in DCM: all deviants tones trials as driving input, and the blocks of active and control light as separate modulatory inputs. Our model included mutual connections between the two regions, self-feedback gain control connections, and the deviant tones as reaching both regions. The model also included the possibility that either light could exert a modulation on both connections between the thalamus and IPS.<br>Time series extracted from individual ROIs were carried into a first-level DCM analysis, in which our model was estimated for each subject. Then, we collapsed the DCMs for a Parametrical Empirical Bayes (PEB) analysis over the first-level DCM parameter estimates. We carried out separate PEB analysis for each matrix (baseline connectivity, effect of driving inputs and modulatory effects) to avoid dilution of evidence effect by reducing the search space. After having estimated the full model (with all connections of interest switched on) for each subject, the PEB approach performs Bayesian Model Reduction (BMR) and Average (BMA) of the parameters across models weighted by the evidence of each model. We then used a threshold based on free energy to evaluate if a parameter contributed to the model evidence. We selected only parameters with strong evidence, meaning with posterior probability (Pp) higher than 0.95. This approach is similar to a p-value ≤ .05 in frequentist statistics (though Bayesian approach do not suffer from multiple comparison issues). Effective connectivity was estimated for the left and the right hemisphere separately.
Effect(s) tested	Modulation exerted by light on the effective connectivity between thalamus and IPS during an auditory oddball task

Specify type of analysis: ☐ Whole brain ☒ ROI-based ☐ Both

Anatomical location(s)	Individual ROI was selected from the resulting statistical map as the first cluster activated in a sphere of 8mm radius centered on the IPS and TH coordinates extracted from the group-level univariate analysis. We also used anatomical landmarks as references for the selection of individual ROIs that were cross-checked using the Juelich Histological Atlas embedded in FSL.

Statistic type for inference<br>(See Eklund et al. 2016)	Univariate analysis : Voxel-wise<br>Effective connectivity: PEB approach
Correction	Univariate analysis: FDR

Models & analysis

n/a	Involved in the study
☐	☒ Functional and/or effective connectivity
☒	☐ Graph analysis
☒	☐ Multivariate modeling or predictive analysis

Functional and/or effective connectivity

DCM gives the rate of change in neural response due to the other neural responses in the system – i.e. the effective connectivity or due to the modulatory inputs (here the light) and is referred to as the bilinear or interaction term.
